# Informal caregivers experience of supplemental oxygen in pulmonary fibrosis

**DOI:** 10.1186/s12955-017-0710-0

**Published:** 2017-07-01

**Authors:** Bridget A. Graney, Frederick S. Wamboldt, Susan Baird, Tara Churney, Kaitlin Fier, Marjorie Korn, Mark McCormick, Thomas Vierzba, Jeffrey J. Swigris

**Affiliations:** 1 Division of Pulmonary Sciences and Critical Care Medicine, University of Colorado Denver, Anschutz Medical Campus, 12700 E 19th Ave, RC2 9th Floor, Aurora, CO 80045-2563 USA; 2 Participation Program for Pulmonary Fibrosis (P3F), P3F Coordinating Center, c/o National Jewish Health, 1400 Jackson Street, F107, Denver, CO 80206 USA; 3 Division of Pulmonary, Critical Care and Sleep Medicine, Sleep and Behavioral Health Sciences Section, National Jewish Health, 1400 Jackson Street, Denver, CO 80206 USA; 4 Autoimmune Lung Center and Interstitial Lung Disease Program, National Jewish Health, Southside Building, Office #G011 1400 Jackson Street, Denver, CO 80206 USA

**Keywords:** Pulmonary fibrosis, Informal caregivers, Oxygen, Interstitial lung disease, Quality of life

## Abstract

**Background:**

Patients prescribed supplemental oxygen (O_2_) therapy face challenges as they adjust to being constantly “tethered” to an oxygen delivery device. Informal caregivers (ICs) of patients with pulmonary fibrosis (PF) face their own, often overlooked hardships when O_2_ is brought into their home and added to their lives. Our aim was to understand the multiple effects of supplemental oxygen therapy on ICs of patients with PF.

**Methods:**

We conducted single, semi-structured telephone interviews with twenty ICs of patients with PF who were using O_2_ for at least 8 months. We performed a qualitative, content analysis based in grounded theory to examine data across subjects.

**Results:**

ICs initially reacted to O_2_ with trepidation and sadness as they came to recognize the changes it would cause in the lives of their patient-loved one (PLO). ICs recognized both beneficial and negative effects of O_2_ on their PLOs. ICs also realized that O_2_ created significant changes in their own lives, including introducing new roles and responsibilities for them, altering their home environments and significantly impacting their relationships with their PLOs. Although O_2_ was a tangible and constant reminder of disease progression, over time ICs were able to adapt and accept their new lives with O_2_.

**Conclusion:**

ICs of patients with PF experience many life changes when their PLO is prescribed O_2_. Having O_2_ prescribers anticipate and recognize these challenges provides an opportunity to give support and guidance to ICs of PF patients who require O_2_ in the hopes of limiting the negative impact of O_2_ on their lives.

**Trial registration:**

Clinicaltrials.gov, registration number NCT01961362. Registered 9 October 2013.

**Electronic supplementary material:**

The online version of this article (doi:10.1186/s12955-017-0710-0) contains supplementary material, which is available to authorized users.

## Background

Pulmonary fibrosis (PF) is an incurable form of interstitial lung disease. In many patients, PF is progressive and induces debilitating dyspnea that, over time, impairs their ability to perform everyday tasks and maintain independence. Patients with PF require supplemental oxygen therapy (O_2_) when they develop hypoxemia (oxyhemoglobin saturation measured by pulse oximetry of ≤89%) either at rest or with activity.

In single-center studies of PF patients, O_2_ has been shown to improve exercise capacity or distance traveled during a timed walk test [1, 2]. Despite its potential physiologic and symptomatic benefits, O_2_ can impair quality of life (QOL) in patients with PF [3, 4] by creating physical challenges (e.g., difficulty moving heavy, unwieldy tanks) and being tethered to tubing which limits patients’ willingness and ability to leave their homes. There are also distressing psychosocial effects – O_2_ is a constant, visible reminder of disease to patients themselves and is perceived as having a “sick person” stigma associated with its use – add to the impairment.

In a previously published study from our group [5], we first heard how O_2_ also creates challenging new realities for informal caregivers (ICs) – family members or friends who provide unpaid assistance to their patient loved-ones (PLOs), helping them to maintain their health, autonomy and independence. ICs are often referred to as invisible second patients [6] due to negative consequences of caregiving on ICs’ physical and psychosocial health. While numerous aspects of providing care affect ICs, little is known about the impact of O_2_ on ICs, particularly in patients with PF. Studies in the COPD literature suggest that ICs are negatively impacted by O_2_ while recognizing some positive benefits O_2_ for their PLOs [7, 8]. Capturing the experiences of ICs of patients with PF is a crucial first step in beginning to address their many unmet needs.

We conducted the current study to learn more about the impact of O_2_ on ICs of patients with PF, specifically (1) to gain an enhanced understanding of the experience of ICs as they are forced to incorporate O_2_ into their lives and (2) to hear ICs’ perceptions about the effects of O_2_ on their PLOs.

## Methods

We recruited a convenience sample of 20 ICs of patients with PF from either the Interstitial Lung Disease Clinic at National Jewish Health (NJH) or using an online strategy through the Participation Program for Pulmonary Fibrosis website to participate in a single, semi-structured telephone interview. Sample size was pre-determined based on similar, prior studies we have conducted, for which saturation was achieved after approximately ten interviews. Saturation was achieved in this study after the 12th interview. Inclusion criteria required that the patient cared for by the IC had been using O_2_ for at least 8 months. All subjects gave written, informed consent. The protocol was approved by the National ewish Health IRB (HS #2790).

All interviews were conducted by members of the research team using an interview guide (Additional file 1) allowing for probing follow-up questions. All interviews were audio recorded and transcribed verbatim. Atlas.ti7 (version 7.5.15; GmbH, Berlin) software was used for data management. Following the completion of all interviews, the analysis proceeded using an iterative process involving established qualitative content methods based in grounded theory [9].

Two analysts (B.A.G. and J.J.S.) independently read the transcripts several times from start to finish to become familiar with the data, achieve immersion and formulate initial impressions. In open coding, each analyst independently developed descriptive codes and applied them to phrases, sentences or larger chunks of data in the transcripts. The analysts met weekly to discuss individual findings and develop consensus around codes. Consensus codes were integrated to develop a thematic framework that encapsulated the data [9]. In the final step, transcripts were reviewed against the backdrop of this framework to fine-tune results and ensure codes and themes captured the essence of the data.

## Results

Participant ICs were predominantly female spouses of their PLOs (Table 1). The average age was just over 62 years, and PLOs had been using O_2_ for an average of nearly 4 years at the time of the interviews. ICs were from various states throughout the United States.

**Table 1:** Demographics of Informal Caregivers

Variable	Result
Female/Male	17/3
Age (range) in years	62.2 ± 9.7 (44–76)
Relationship to Patient	16 Spouses, 4 Children
State of residence	2 CA, 4 CO, 2 WA, and 1 each from FL, GA, ID, IL, MD, MN, NE, NM, NY, OH, PA, UT
Duration of O_2_ use (years)	3.9 ± 3.0
O_2_ set-up	
In-house	19 home concentrator, 1 liquid oxygen
Portable	9 compressed gas only 3 liquid oxygen only 4 POC only 3 compressed gas and POC available 1 liquid oxygen and POC available

*Abbreviations*: *O*_*2*_ supplemental oxygen, *POC* portable oxygen concentrator, *CA* California, *CO* Colorado, *WA* Washington, *FL* Florida, *GA* Georgia, *ID* Idaho, *IL* Illinois, *MD* Maryland, *MN* Minnesota, *NE* Nebraska, *NM* New Mexico, *NY* New York, *OH* Ohio, *PA* Pennsylvania, *UT* Utah

Four primary themes emerged from caregivers’ experiences with O_2_: (1) ICs’ initial reactions to O_2_, (2) perceived impact of O_2_ on PLOs, (3) O_2_-induced life changes for ICs and (4) ICs’ adaptation to and acceptance of O_2_ (Table 2).

**Table 2:** Four Major Themes and their Sub-Themes

Themes and Sub-themes	Illustrative Quotations
Initial Reactions to O_2_ Making PF real Overwhelmed Worry and fear Gratitude	*“It isn’t a cure, because there is no cure, but it [O*_*2*_ *] is the next best thing.”*
Effect of O_2_ on PLOs Adverse physical effects Beneficial physical effects Emotional effects Tethered	*”It’s maintaining her and, uh, for that I’m grateful. And I saw a lot of confidence when I gave her the portable oxygen machine initially. She was super-excited about it; you could tell that she felt good. She felt confident.”*
Life changes for ICs Impositions of O_2_ Emotional response Role change	*“Well, no -- because, um, you know, any time you have, um, anything to change it so drastically in your life like that, you know, everything changes. You, you, um, you can’t, um, you know, you have to, you have to work, work everything in your life around the oxygen. You, you, can’t, um, you know, you can’t just up and go. You can’t just, uh, um, you know, do -- there’s certain things you can’t do. You know. It’s just, it’s changed life a lot, but definitely not for the better.”*
Adaptation and Acceptance Dealing with practical limitations Relationship changes Leaving home Tactics to achieve acceptance	*“It didn’t take long for me to get to the point of, well, if it’s going to help you, let’s go for it, and let’s do whatever we need to do.”*

### ICs’ initial reactions to O_2_

When O_2_ was first prescribed, it evoked strong emotional reactions. Many ICs recalled being “devastated” or “shocked.” It was a time when “everything change[d]” for them and their PLOs – “one of our most depressing days.” Suddenly, ICs found themselves concerned about things like if the electricity went out, if the O_2_ would explode or cause a fire in the home, or if they had enough O_2_ to maintain adequate blood oxygen if they left home for extended periods of time.

Some felt “sad” or “bad” for their PLO, because “[O_2_] is part of his life now.” The daughter of a patient who had been using O_2_ for 2 years recalled how “vulnerable” needing O_2_ made her mother seem to her. O_2_ was an “in your face” reminder that “meant he was sick”; an ever-present “…thing that just sits there all the time…her need for oxygen.”

A few ICs reacted more positively to O_2_. The daughter of a PF patient on O_2_ for 2 years mentioned that she was “not as worried about her [mother]” after she was prescribed O_2_. Others felt “reassured” by their PLO having O_2_, viewing it as a safety net if he “gets into trouble.”

### Perceived impact of O_2_ on PLOs

ICs noted both beneficial and adverse effects of O_2_ on their PLOs. ICs generally felt that O_2_ made PLOs “feel better.” The comment from a husband of a patient on O_2_ for 3 years captures the positive aspect of the theme best: “…with the oxygen it makes it better. Not perfect, but better.” ICs perceived O_2_ as allowing PLOs to be more active: several resumed activities they had once enjoyed but had given up before starting O_2_. ICs also noted improvements in fatigue, energy levels, morning headaches and anxiety. To ICs, PLOs looked “more comfortable” when using their O_2_: they were visibly less short of breath and had “pink cheeks” or “color in his face” – things they didn’t have in the months before O_2_.

### ICs also saw how O_2_ was limiting

*“It limits her—uh, her, in where she can go, how long she can go, how far she can go.”* [Subject #2]

Inside their homes, PLOs were viewed as “tied,” “tethered” or on a “leash”. Cannulas frequently got stuck on something (e.g., furniture) and for some, were not long enough to allow patients to reach certain areas of the home. Outside the home, ICs saw how PLOs had to “drag” or “lug” their O_2_ around with them. Some reported how PLOs thought their O_2_ delivery device was too noisy, so they avoided going to public places, because they didn’t want to disturb other people. This usually meant ICs didn’t go either.

ICs recognized how O_2_ caused psychosocial challenges for PLOs. Most notably, O_2_ became a constant, tangible reminder to PLOs they were sick; it made them “feel very frail and fragile.” O_2_ made PLOs self-conscious; for some, to the point of not using it when needed, due to fears of stigmatization.

The wife of one patient commented, *“He would not, um, you know, be caught dead walking around [grocery store name] hauling a tank behind him.”* [Subject #4]

It also affected important social interactions: as the wife of a patient on O_2_ for 18 months recalled a time when her husband was using a portable oxygen concentrator, and their 3-year-old granddaughter *“came up to him and she goes, ‘Pops, run with me!’ and he says, ‘Well, I can’t. I need to, you know…I have to have this [O*_*2*_ *].’”* [Subject #3]

### O_2_-induced life changes for ICs

For most ICs, the addition of O_2_ in the house meant extra physical duties related to O_2_, including filling and/or carrying tanks, loading tanks in the car prior to leaving home, helping care for and clean equipment, and making sure things were ordered on time. Typically, duties were met without resistance.

For some ICs, including the husband of a patient who rapidly declined and died after less than a year on O_2_, things were more challenging*, “It was a 24/7 job that consumed all my time…I didn’t think about doing anything but maintaining…maintaining her oxygen.”* [Subject #20]

The wife of a patient who had been on O_2_ for 9 years summed it up thusly*: “…you have to work, work everything in your life around the oxygen…it’s changed [my] life a lot, but definitely not for the better.”* [Subject #15]

Once O_2_ was brought into the home, ICs were also relegated to literally (and figuratively in many cases) doing the heavy lifting around the house. They often took on chores that required physical exertion, even when PLOs could probably have managed them – but only after increasing their O_2_ flow. ICs did not want their PLOs to “exert extra energy.” And they fell into the role fairly easily – as one IC mentioned, “I don’t know; I mean, it’s my job, you know?”

Most ICs also took on the job of trying to ensure their PLOs used O_2_ as prescribed. Particularly early on, they would “nag” or “prod” patients to put their cannulas on or turn up the flow. As one IC explained, “you ride a fine line between how firm do you get, you know, and how soft do you stay, to get accomplished what you feel is important.” PLOs would sometimes heed their IC’s advice and other times “totally ignore it.”

O_2_ caused significant changes to ICs relationships with their PLO: O_2_ “slowed [them] down.” It forced a literally slower pace and placed unwanted limitations on several aspects of their lives. Spontaneity was no longer an option. Some ICs were silently “frustrated” because their PLOs could not “keep up.” Most pairs, even those in whom the PLOs used a portable oxygen concentrator (POC) and could, curtailed traveling.

Leaving home took extra time and planning – greater oxygen needs required more thought and preparation. “You can’t just fly out the door,” said the wife of a patient on O_2_ for 18 months. Another IC recalled, “Everything was an expedition if we were going out.” Before leaving, they had to “figure out what your [O_2_] needs are going to be while you’re gone.” Leaving the home with O_2_ was like “running errands with a baby” (packing up diapers, bottles, clothing, etc.).

For pairs in which PLOs required high-flow systems (i.e., at least six liters per minute continuous flow), out-of-home activities were particularly constrained. The wife of a patient told how she and her husband brought their dog of 11 years to the veterinarian’s office to be euthanized, but devastatingly, they had to leave before the dog was put down, because the patient’s oxygen was going to run out.

### Adaptation and acceptance of O_2_

When O_2_ was first prescribed, ICs and PLOs found themselves attempting to simultaneously digest what the need for O_2_ was telling them about disease status (it was serious); trying to educate themselves about some of the practical issues around O_2_ (like learning what equipment they needed and figuring out how to navigate the system to get things in a timely fashion); and finding motivation to begin the process of “embracing the new normal.”

ICs of patients with progressive disease were required to re-accept and re-adjust as pulmonary fibrosis worsened and oxygen needs increased. As one IC stated, “Before he was on it 24/7, it didn’t seem like a big deal.” ICs of patients who used O_2_ 24/7 but whose needs could be met with pulsed flow (from either a POC or via home-fill compressed gas tanks) faced challenges, complexities and constraints but seemingly not as many as ICs of patients who required continuous high-flow O_2_.

*“And then when the guy came with the higher flow, he realized he couldn’t carry his oxygen and knew that his mobility would be cut down even further.”* [Subject #9]

For most ICs, the prospect of things worsening was ever-looming.

*“You’re kind of looking down the road…It -- how long we will be able to continue to do this [use his pulsed flow regulator], and so it -- I mean, it changes your lifestyle, that’s for sure.”* [Subject #16]

*“I know at some point it’s probably not going to be enough oxygen [delivered via the POC], and we probably will end up going back to tanks.”* [Subject #5]

*“When I think about people who have much higher oxygen needs than he does, that the chance of them leaving the house would be slim. And that’s, you know, that’s a little frightening.”* [Subject #5]

ICs saw how O_2_ induced great uncertainty for their PLOs, representing a “major change in [their PLOs’] health status and [left ICs questioning] how I’m going to be living my [own] life” – particularly if things continue to worsen. Over time, ICs accepted and adapted to the change and developed strategies for living.

The husband of a patient who had been using O_2_ for 2 years recalled thinking, *“She needs it, so this is going to be a part of our life, and I’m not going to worry about it.”* [Subject #18]

But, getting there took time and effort. ICs used several methods to cope, adapt and “deal with whatever needs to be dealt with.” A wife of a patient on O_2_ for 2 years commented, “It was hard at first, just getting used to it, just the adjustment period at first.” It was clear that ICs and PLOs worked together and relied on each other to adapt to the new way of life with O_2_. The wife of a patient on O_2_ for 9 years said they dealt with the challenges of O_2_ with “lots of love.”

IC/PLO pairs had to find balance in their lives: it was impossible for them to completely disregard the presence of O_2_, but they realized they desperately wanted and needed to continue to live. The leash and limitations never left; they just learned to “not fight it,” “relax into it” and deal better with them – emotionally and physically.

*“He has to live too, so it’s balancing things out.”* [Subject #8]

There was a strong motivation by some dyads to maintain a sense of normalcy in their lives by getting out, remaining socially active, “enjoy[ing] every day,” “enjoy[ing] life as much as you can.” A wife of a patient on O_2_ for 3 years said, “Well, we don’t ever think of it as good or bad; it just is what it is, and you deal with it.” Putting forth a conscious effort to maintain a positive outlook was vital. Some mentioned that keeping a sense of humor, laughing and joking were important as they moved toward acceptance of O_2_.

Acceptance required they “change the way you do things” or “taking baby steps” to prove to themselves they could still live their lives as a couple. They – often subconsciously – adjusted how things were done around the home. One IC mentioned doing the cooking and her PLO did the preparing (e.g., chopping, mixing) of food so the O_2_ would not be near the stove. Ultimately, most ICs found that dealing with O_2_ eventually became “so routine and so much a part of—of the way we live now.”

## Discussion

We interviewed 20 ICs of patients with PF to learn more specifically about the effects of O_2_ on ICs, patients and the IC/patient relationship. Similar studies were conducted on ICs of patients with COPD [7, 8], but to our knowledge, this is the first study to focus on the impact of O_2_ on ICs of PF patients.

Although O_2_ is a vital therapy for many PF patients, it commands significant changes in their lives and in the lives of their ICs. ICs reminded us that an O_2_ prescription for a patient is really a prescription for the IC, too; in fact, it is a prescription for the entire home. Although patients wear the cannulas and wrangle with the O_2_ delivery devices, ICs cannot escape the reaches of O_2_.

With a PLO who uses O_2_, ICs were forced to accommodate a noisy concentrator and tubing throughout the home, to accept new worries about equipment and the O_2_ supply, to take on new roles, and to live with the constraints of having a patient-partner who was unable or unwilling to be as physically or socially active as they once were. And, they had to do all these after working through the initial shock and sadness of their PLO being prescribed O_2_ – and while being constantly reminded of their PLO’s potentially progressive, terminal illness.

Aiming to improve understanding of what life is like for patients with idiopathic pulmonary fibrosis (IPF) and their family caregivers, Overgaard and colleagues [10] conducted interviews with 24 patient/IC dyads. The experiences of ICs they heard about were similar to what we heard. Overgaard et al. found that O_2_ created role changes for ICs, who became responsible for the physical duties related to oxygen and took over chores that were once completed by their PLOs. Like us, they heard how O_2_ was a tangible, visible sign of “deterioration” and how O_2_ altered the home environment. Patients and ICs also described a phenomenon we have labeled the “Shrinking World Syndrome” that affects both members of the dyad [5]. This is when ICs and their PLOs become confined to a “smaller radius of action.” As PLOs become increasingly constrained by O_2_ and unable (or unwilling) to leave the home, ICs’ worlds shrink—their social interactions decline and certain relationships fall by the wayside. In the current study, we extend the findings of Overgaard and colleagues, by among other things, observing the syndrome was particularly apparent when a portable oxygen concentrator could not provide high enough oxygen flows to maintain normal oxygen saturations, thus forcing patients to use bulkier, heavier O_2_ delivery devices whose O_2_ supply did not last long.

Results from studies of the impact of O_2_ on ICs of patients with COPD are similar: ICs are forced to accept new roles as they attempt to navigate from fear and anger to acceptance—a process that mirrors the grief cycle [8]. One striking difference in studies of O_2_ in COPD is that O_2_ was more consistently reported to have beneficial effects for PLOs including increased social participation, increased quality of life and improved ability to leave the home [7].

Despite the hardships associated with O_2_, with time, the majority of ICs developed a begrudging acceptance of it as a permanent element of the new normal. They achieved this acceptance by focusing on the beneficial aspects of O_2_ and gradually, often subconsciously, making small changes – around the home, in their daily routines, and in their perspectives – until O_2_ was simply part of life.

There are limitations to this study. PLOs had various forms of PF, so this may limit the applicability of the findings to any one specific type of PF. However, based on the data from this and prior studies from our group, we believe the perceptions and experiences around O_2_ are similar regardless of PF etiology. That subjects hailed from 15 different states protects from single-center bias.

## Conclusions

Supplemental oxygen therapy affects the lives of patients with PF and their caregivers. Caregivers see, first-hand, how O_2_ can help—and create hardships—for their PLOs. O_2_ also affects caregivers’ lives as they gradually come to accept the new normal: it alters their home environments, creates new responsibilities for them, limits their interactions with the outside world and influences their relationships with their PLOs.

Because O_2_ is ubiquitous in the therapeutic regimens of patients with progressive PF, and ICs provide a significant amount of their everyday care, we must strive to better understand ICs’ perceptions. Having an improved appreciation of their experiences with O_2_ is a first step in the process of developing interventions aimed at improving quality of life and decreasing burden for ICs of patients with PF.

## Additional file

Additional file 1: Appendix to Informal caregivers experience of supplemental oxygen in pulmonary fibrosis. (DOCX 15 kb)

## Abbreviations

IC: Informal caregiver
IPF: Idiopathic pulmonary fibrosis
O_2_: Supplemental oxygen therapy
PF: Pulmonary fibrosis
PLO: Patient-loved one
QOL: Quality of life
