# Supplementary material for: Informal caregivers experience of supplemental oxygen in pulmonary fibrosis
Source: Health Qual Life Outcomes. 2017 Jul 1;15:133. doi: 10.1186/s12955-017-0710-0 (PMC5494136; doi:10.1186/s12955-017-0710-0)
Supplement: Additional file 1: — Appendix to Informal caregivers experience of supplemental oxygen in pulmonary fibrosis. (DOCX 15 kb) [file 12955_2017_710_MOESM1_ESM.docx]

**Appendix to Informal caregivers experience of supplemental oxygen in pulmonary fibrosis**

Bridget A. Graney, MD^1,2^, Frederick S. Wamboldt, MD^2,3^, Susan Baird^2^, Tara Churney, MPH^2,4^, Kaitlin Fier, MPH^2,4^, Marjorie Korn^2^, Mark McCormick^2^, Thomas Vierzba^2^, Jeffrey J. Swigris, DO, MS^1,2,3,4^

^1^Division of Pulmonary Sciences and Critical Care Medicine, University of Colorado Denver, Anschutz Medical Campus, 12700 E 19^th^ Ave, RC2 9^th^ Floor, Aurora, CO 80045 USA

^2^Participation Program for Pulmonary Fibrosis (P3F), P3F Coordinating Center, c/o National Jewish Health, 1400 Jackson Street, F107, Denver, CO, 80206 USA

^3^Division of Pulmonary, Critical Care and Sleep Medicine, Sleep & Behavioral Health Sciences Section, National Jewish Health, 1400 Jackson Street, Denver, CO, 80206 USA

^4^Autoimmune Lung Center and Interstitial Lung Disease Program, National Jewish Health, Southside Building, Office #G011 1400 Jackson Street Denver, CO 80206, USA

**Informal Caregiver Interview**

*Interviewer (I):* Hello Mr./Mrs./Ms. _______, how are you doing today? My name is _______ and during this phone interview I will be asking you some questions on your perspective of supplemental oxygen. Does this sound like something you’d be comfortable discussing today?

*Primary*

*Supporter (IC):* ____________________________________________________________

*I:* [if response positive]: Great! Please let me know, however, if you become uncomfortable at any point during the discussion.

[if response negative]: Is there anything I can do to make you more comfortable discussing this topic with me?

*IC:* ____________________________________________________________

*I:* [if applicable]: Ok, I will try to do that/keep that in mind.

[to everyone]: All right, before we get started, I want to make sure you’ve had the chance to observe somebody who uses supplemental oxygen. If so, would you mind telling me what your relationship is with this person?

*IC:* ____________________________________________________________

*I:* Are you comfortable with me referring to this person as ________?

*IC:* Great, let’s get started. How long has _______ been using supplemental oxygen?

*IC:* ____________________________________________________________

*I:* Do you know what kind of set-up or delivery system ________ uses when he/she is at home and outside of the home?

*IC:* ____________________________________________________________

*I:* Would you say _____ uses oxygen in the way it is prescribed? In other words, does he/she use oxygen as much as he/she is supposed to?

*IC:* ____________________________________________________________

*I:*  [if yes]: Is it easy for him/her to use oxygen as much as he/she is supposed to?

[if no]: Why do you think he/she doesn’t use oxygen as much as he/she is supposed to?

*IC:* ____________________________________________________________

*I:* How about at night? Do you know if _____ is able to keep his/her oxygen on throughout the night? That is assuming he/she is supposed to wear it at night….

*IC:* ____________________________________________________________

*I:* Do you find that you or _______ has to plan your or his/her day around his/her oxygen use?

*IC:* ____________________________________________________________

*I:* Ok, now I’d like you to think about how things have changed for _____ since he/she started using oxygen. Have you seen any improvements in the way she/he feels?

*IC:* ____________________________________________________________

*I:* Are there any other improvements in _____’s life that you can think of?

*IC:* ____________________________________________________________

*I:* How about in your life? Have things changed for the better for you at all since ______ went on oxygen therapy?

*IC:* ____________________________________________________________

*I:* Ok, we covered some good things about supplemental oxygen. Now I’d like to ask what ­_______ doesn’t like about using oxygen.

*IC:* ____________________________________________________________

*I:* Is there anything else you can think of that _______ doesn’t like about oxygen?

*IC:* ____________________________________________________________

*I:* How about you? Are there things you don’t like about ______ using oxygen?

*IC:* ____________________________________________________________

*I:* Thinking about the things you and ______ don’t like about oxygen… can you tell me how you both deal with these issues/problems?

*IC:* ____________________________________________________________

*I:* Are there things you wish could be different about ________ having to use oxygen?

*IC:* ____________________________________________________________

*I:* In a similar regard, are there other ways in which you feel _______’s oxygen use has changed things in either or both your lives?

*IC:* ____________________________________________________________

*I:* Ok, now I’d like to ask you to think about before _____ went on oxygen. Did either of you know much or anything about supplemental oxygen at that point?

*IC:* ____________________________________________________________

*I:* Did you know anyone who used oxygen?

*IC:* ____________________________________________________________

*I:* Do you remember how you both *felt* about oxygen?

*IC:* ____________________________________________________________

*I:* [if not already addressed]: Do you remember either of you feeling scared about ______ using oxygen?

*IC:* ____________________________________________________________

*I:* [if not already addressed]: Were there any other emotions you can think of that either or both of you experienced regarding oxygen use?

*IC:* ____________________________________________________________

*I:* Thinking about those feelings and where both of you are at now, would you say your thoughts about oxygen were justified?

*IC:* ____________________________________________________________

*I:* Was there anything about using oxygen that surprised you, in either a good or bad way?

*IC:* ____________________________________________________________

*I:* How about your relationship with _______. Has that been affected by ______’s oxygen use?

*IC:* ____________________________________________________________

*I:* Has this had an effect on you emotionally and/or mentally? Having to help ______ with his/her oxygen use?

*IC:* ____________________________________________________________

*I:* In what other ways has this change affected your life? Either independently or together with _______.

*IC:* ____________________________________________________________

*I:* Are there any resources you or _______ use to help you deal with _______’s oxygen use?

*IC:* ____________________________________________________________

*I:*  Is there anything you can think of that might help you both live with ________’s oxygen use?

*IC:* ____________________________________________________________

*I:* Do you have any advice for people who will make a similar transition to using oxygen as you and ________ have?

*IC:* ____________________________________________________________

*I:* Ok, before we wrap up, I’d like to ask if there’s anything you think we didn’t cover today as far as your thoughts and feelings on supplemental oxygen?

*IC:* ____________________________________________________________

*I:* Great, thank you. We are finished with the interview now. I just want to thank you again for your time and participation. I appreciate you sharing your thoughts with me, especially on those tougher questions. It’s been a pleasure speaking with you and I hope you have a great rest of your day.
